# Resurfacing with Ablation of Periorbital Skin Technique: Indications, Efficacy, Safety, and 3D Assessment from a Pilot Study

**DOI:** 10.1089/pho.2018.4479

**Published:** 2018-10-05

**Authors:** Stefania Guida, Steven Paul Nisticò, Francesca Farnetani, Ester Del Duca, Nathalie De Carvalho, Flavia Persechino, Tommaso Verdina, Luca Giannetti, Martina D'Alessandro, Giacomo Giovanni Urtis, Giovanni Pellacani, Giovanni D'Alessandro

**Affiliations:** ^1^ Dermatology Unit, Department of Surgical, Medical, Dental and Morphological Science with Interest transplant, Oncological and Regenerative Medicine, University of Modena and Reggio Emilia, Modena, Italy.; ^2^ Dermatology Unit, Department of Health Sciences, University Magna Graecia, Catanzaro, Italy.; ^3^ Dermatology Division, Department of System Medicine, University of Tor Vergata, Rome, Italy.; ^4^ Institute of Ophthalmology, University of Modena and Reggio Emilia, Modena, Italy.; ^5^ Pedodontics Division, Department of Surgical, Medical, Dental and Morphological Sciences with Interest transplant, Oncological and Regenerative Medicine, University of Modena and Reggio Emilia, Modena, Italy.; ^6^ School of Medicine, University of Verona, Verona, Italy.; ^7^ Dr Urtis Clinic, Milano, Italy.; ^8^ Istituto di diagnosi e terapie dermatologiche ed estetiche (ISDET), Bari, Italy.

**Keywords:** lower eyelid, CO_2_ laser, resurfacing

## Abstract

***Objectives and background:*** Nowadays, several approaches for skin rejuvenation of the lower eyelid are available. We present a new technique of resurfacing with ablation of periorbital skin (RAP) performed in a single session. ***Methods:*** This is a retrospective study involving 20 patients showing skin elastosis with or without evidence of the nasojugal fold and atrophic and dyschromic skin or needing a combined approach of transconjuntival lower blepharoplasty for fat bag removal. RAP technique is assessed in terms of efficacy, safety, and 3D evaluation of results at 6 months' follow-up.

***Results:*** Global Assessment Improvement Scale results highlighted an improvement in all cases, for both physicians (blinded to treatment) and individual subjects. Only minor adverse events (edema, erythema, and discoloration) were reported in almost all patients, lasting 2–3 weeks after treatment, and were resolved without intervention. A 3D imaging tool revealed the reduction of medium protrusions and depressions and an improved texture at 2 months. Skin recovery was inversely correlated with hemoglobin reduction.

***Conclusions:*** RAP seems to offer expert dermatologists a safe and clinically effective technique for skin rejuvenation of lower eyelids, without significant adverse events. Further studies will be performed to confirm our results.

## Introduction

The periorbital region is one of the areas showing the earliest signs of aging. Skin around the eyes presents special features that should be considered when treating this region. Among these features are smoothed dermal–epidermal border, thin dermis, and low density of sebaceous glands.^1^ Further, a thin and superficially located subcutaneous tissue is present and specific skin folds are created as a result of overactivity of the orbital part of the *orbicularis oculi* muscle. For these reasons, the periorbital skin is characterized by low elasticity, is prone to skin wrinkles, discoloration, and the formation of fat bags under eyes.^2^

While fat bags require surgical intervention, several options are available for periorbital skin rejuvenation.^3,4^ Carbon dioxide (CO_2_) laser is a laser device producing energy in the far infrared, using a wavelength of 10,600 nm. Resurfacing with CO_2_ laser has been used in the treatment of scars and skin aging, as well as for unconventional applications.^5–7^ Nevertheless, ablative CO_2_ laser treatment requires general anesthesia or sedation, long-lasting recovery, and can induce potential adverse events.^8,9^ However, the high efficacy of ablative CO_2_ laser makes it a powerful weapon in the treatment of many skin conditions by experienced hands.^5^ Thus, we present herein a new technique combining the efficacy of freehand CO_2_ ablative resurfacing with topical anesthesia, short downtime, and high safety profile. No previous data are available for the use of freehand CO_2_ laser resurfacing with ablation of periorbital skin (RAP). This study aims to assess the application of the new RAP technique for lower eyelid skin rejuvenation in terms of efficacy (three-dimensional, operator, and patient assessments) and safety (adverse events).

## Patients and methods

### Population

**FIG. 1.**
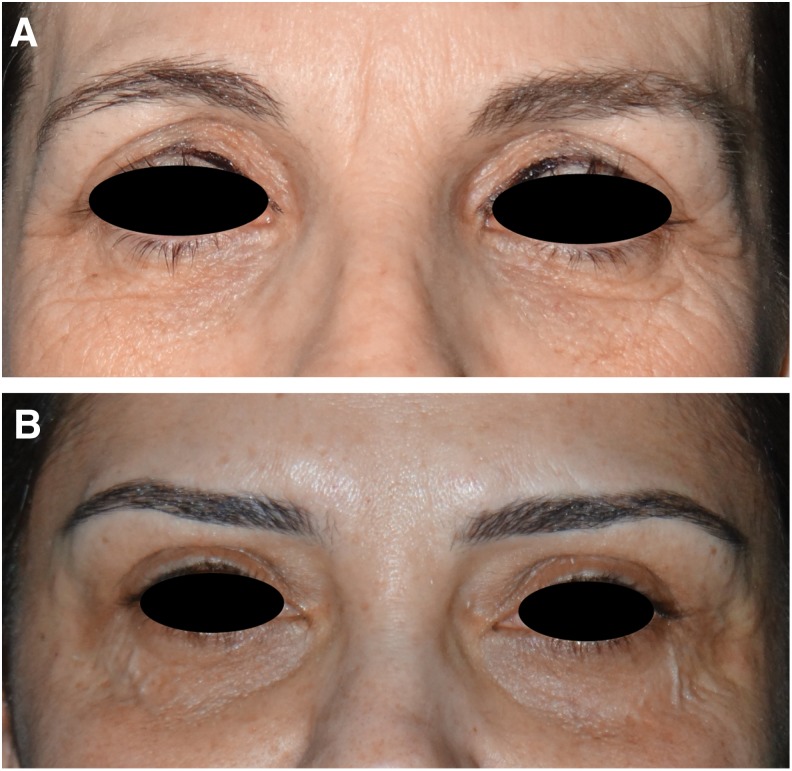
Indications for treatment: **(A)** skin elastosis and **(B)** atrophic skin with heterogeneous color skin.

This retrospective study analyzed lower eyelid rejuvenation in 20 consecutive patients treated with the freehand CO_2_ laser technique. Three of the total 20 patients were treated with a combination of transconjuntival lower blepharoplasty for fat bag removal and RAP technique. Inclusion criteria for treatment included skin phototypes I–III and the presence of moderate lower eyelid laxity (skin elastosis with or without evidence of the nasojugal fold), according to the Facial Laxity Rating Scale,^10^ in combination with atrophic and dyschromic skin (Fig. 1). Exclusion criteria for treatment included previous keloid scarring, a history of connective tissue disease, oral retinoids within 6 months before the treatment, any kind of treatment for the lower eyelid within 1 year before the study, pregnancy, immunosuppressive drug assumption, and any other disease affecting the wound healing process. All patients were asked to apply an SPF50+ sunscreen and to avoid UV exposure for 30 days before the laser session.

All procedures performed in studies involving human participants were in accordance with the Helsinki Declaration and its later amendments or comparable ethical standards. All patients provided informed written consent.

### Laser device

All patients were treated by a single investigator (G.D.), with the SmartXide2 CO_2_ 60 W (DEKA-M.E.L.A., Calenzano, Italy) laser device, in a private practice *scenario*.

**FIG. 2.**
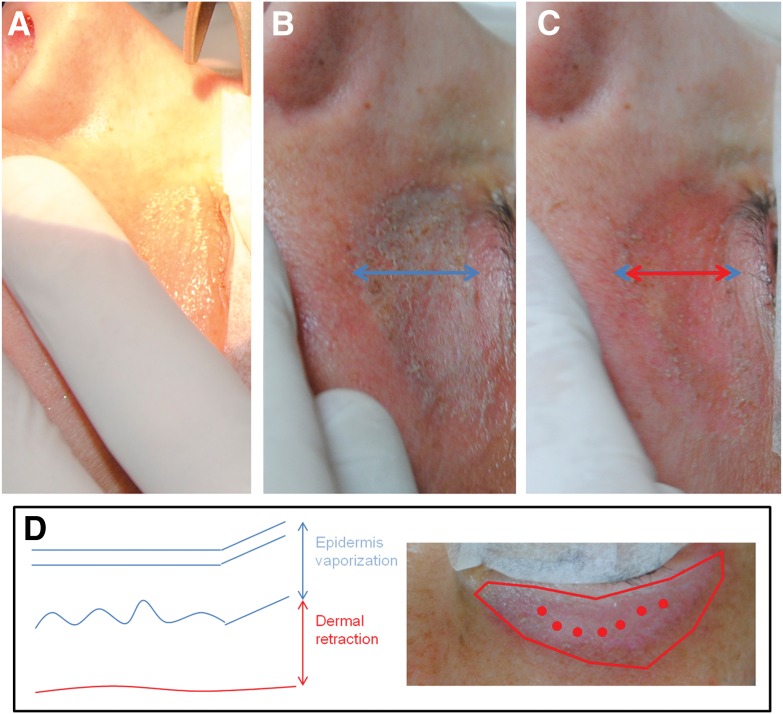
Intraoperative pictures: **(A)** removal of topical anesthesia, **(B)** epidermis vaporization (maximum vertical diameter in blue) corresponding to opalescent skin associated with microvesicles, and **(C)** dermal retraction (maximum vertical diameter in red) obtained after defocused irradiation of the dermis. **(D)** Schematic representation of how a dermal retraction can be performed. Following traction lines, a centripetal retraction is obtained with single or linear pulses. The sum of each pulse will determine the total amount of retraction.

The SmartXide2 system is a CO_2_ laser with 60 W of maximum power, enabling emission of high energies in pulsed modes. In detail, the PSD^®^ (Pulse Shape Design) technology permits precise action on selected layers, inducing minimal thermal damage. In this study, the Smart Pulse modality, allowing precise vaporization of targeted areas, was employed. Therefore, based on the selective photothermolysis principle, selecting the wavelength, fluence, and pulse duration, the desired target can be destroyed without damaging the surrounding tissue. Clinically, color indicators of the skin visually guide the assessment of selective layer vaporization; the skin becomes opalescent with microvesicles (Fig. 2B). These clinical aspects have been previously associated (at histology) with the detachment of the epidermis from the dermis.^11^

### Procedure

The skin was prepared with a disinfecting agent and then a topical anesthetic was applied to the treatment area. As the target of the CO_2_ laser is the water of the skin, an anhydrous cream, comprising lidocaine 10% in mineral oil and polyethylene, was applied. Adequate eye protection was provided to patients during the treatment.^12^

Complete vaporization of the epidermis was performed according to a freehand motion and focused radiation, covering the entire treatment area in a homogeneous manner (Fig. 2). Settings used were power 0.5 W, energy 50 mJ, frequency 10 Hz, and energy density 150–250 J/cm^2^ for a total of 300–500 sec. Vaporized skin was not removed to avoid too superficial or too deep vaporization.

The same parameters used for the epidermis were employed to obtain a dermal retraction. In detail, 0.5 W power, 50 mJ energy, single pulse, and defocused irradiation were applied. The skin visibly retracts following a single pulse, so the distribution (linear or circular) was used depending on the area and amount of retraction observed (Fig. 2).

A single session of treatment was performed for all patients.

### Postoperative procedure

The postoperative regimen consisted of medication with petroleum jelly for 7–10 days. After that patients were instructed to use an antibiotic ointment twice a day for 1 week, followed by a moisturizer (Toleriane Ultra Fluid^®^, La Roche Posay, France) once a day for 3 weeks.

### Efficacy

The efficacy of freehand CO_2_ laser treatment for the lower eyelid was evaluated by two external dermatologists (S.G. and F.F.) who analyzed images at baseline and 6 months post-treatment. For each subject, dermatologists were asked to identify which was the post-treatment image.

Efficacy was also evaluated through the Global Aesthetic Improvement Scale (GAIS) completed by both the physician/investigator (PGAIS) and subjects enrolled (SGAIS) at the 6th month post-treatment visit. Additionally, a 3-point satisfaction questionnaire (very satisfied, satisfied, and not satisfied) for patients was also administered.

### Safety

Before treatment, the medical history of each subject was analyzed and a physical examination of the area to be treated was performed. Subject pain assessment was made during treatment using a validated 10-point (1–10) Numeric Rating Scale, where 1 indicated no pain and 10 was referred to as the worst possible pain. The treated area was examined ∼30 min after treatment for evidence of erythema or edema or ecchymosis. At each subsequent visit, subjects were asked about possible adverse events, which were classified according to minor events, not requiring additional interventions or medications, or major events, including events requiring additional interventions or medications, scarring, and permanent hyperpigmentation.

### 3D assessment

A novel device called Antera 3D (Antera 3D; Miravex, Limited, Dublin, Ireland) was used for *in vivo* optical skin imaging to analyze optical skin structure and confirm the clinical response of the treatment. This device exploits multi-directional illumination and computer-aided reconstruction of skin surfaces and evaluates distribution and concentration of hemoglobin.^13^ Images were obtained before treatment at 7 days and at 1, 2, and 3 months post-treatment. We assessed a medium degree of depression, texture, and protrusion to estimate results and hemoglobin levels, related to inflammation, to estimate recovery time after the procedure. A full 3D reconstruction of the target area was also provided.

## Results

A total of 20 female patients were enrolled. The mean patient age was 46.6 ± 5.8 (range, 35–57). An additional lower transconjuntival blepharoplasty procedure for fat bag removal was performed in three cases.

### Efficacy

**FIG. 3.**
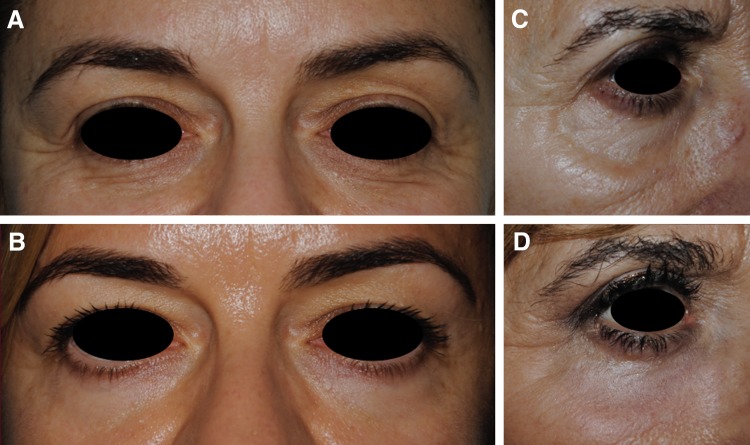
Clinical picture of a 45-year-old woman **(A)** pretreatment and **(B)** 6 months post-treatment; clinical picture of a 52-year-old woman **(C)** pretreatment and **(D)** 6 months post-treatment.

According to the initial clinical images and those obtained at 6 months, the blinded external dermatologists correctly identified the images of pre- and post-treatment (Fig. 3). Both the treating dermatologists and the patients themselves reported, overall, an all improved score according to the GAIS scales (Table 1). In detail, patients recorded “very much improved” in 75% of cases and dermatologists in 65%. Further, the esthetic satisfaction evaluation revealed that 80% of patients were very satisfied and the other 20% were satisfied.

**Table 1.** Global Aesthetic Improvement Scale Scores at Month 3

*PGAIS,* N *(%)*	
Very much improved	13 (65)
Much improved	6 (30)
Improved	1 (5)
No change	0
Worse	0
All improved	20 (100)

*SGAIS,* N *(%)*	
Very much improved	15 (75)
Much improved	5 (25)
Improved	0
No change	0
Worse	0
All improved	20 (100)

PGAIS, Physician Global Assessment Improvement Scale; SGAIS, Subject Global Assessment Improvement Scale.

### Safety

Mean pain score was 3.7 ± 1.2 (range, 2–5) on a 10-point scale.

Three adverse events were reported: erythema, discoloration, and edema. These were observed after the procedure in almost all patients, lasted from 2 to 3 weeks, and were resolved without additional treatment. No major events were reported.

### 3D assessment

**FIG. 4.**
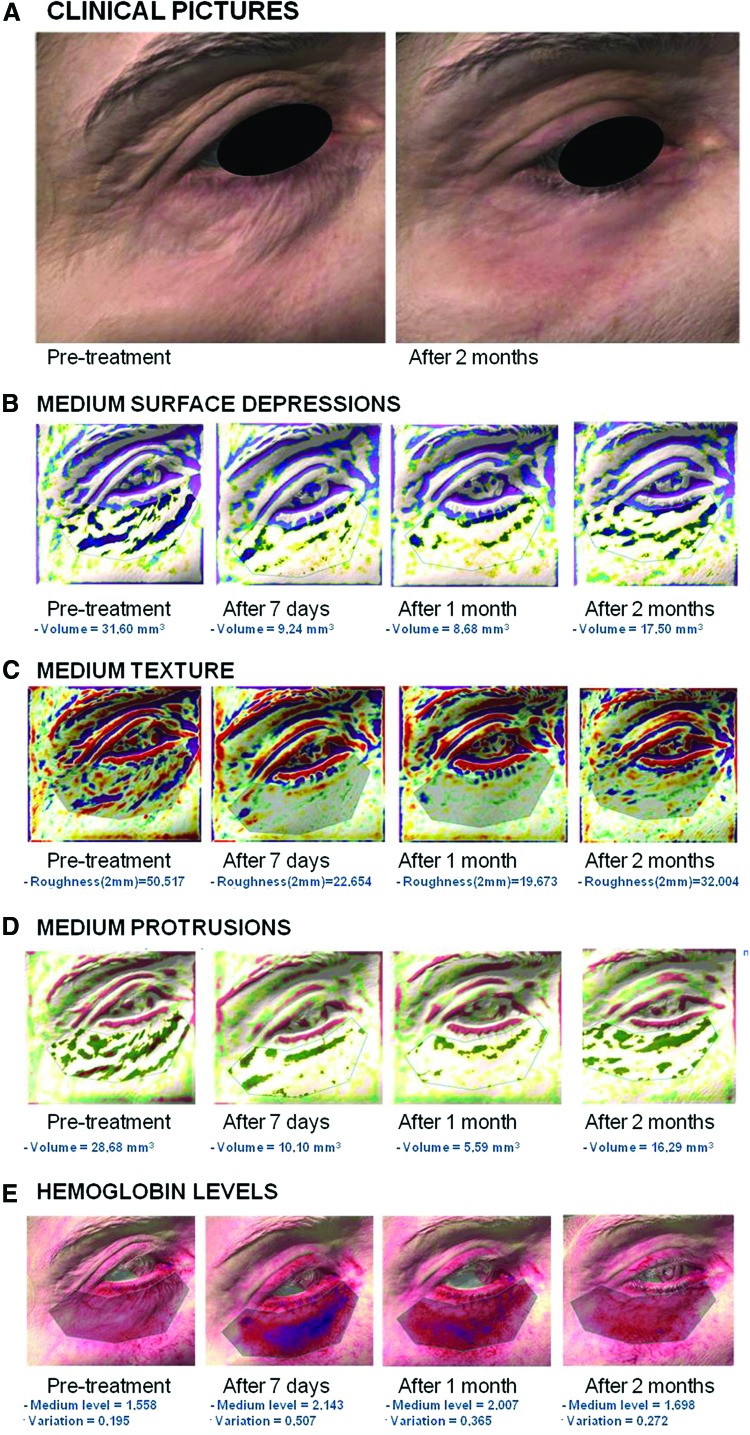
Antera 3D pictures: (**A)** pretreatment and two months post-treatment of a 45-year-old woman. **(B)** Medium surface depressions at different time intervals: pretreatment, 7 days, 1 month, and 2 months post-treatment. **(C)** Medium texture at different time intervals: pretreatment, 7 days, 1 month, and 2 months post-treatment. **(D)** Medium protrusions at different time intervals: pretreatment, 7 days, 1 month, and 2 months post-treatment. **(E)** Hemoglobin at different time intervals: pretreatment, 7 days, 1 month, and 2 months post-treatment.

**FIG. 5.**
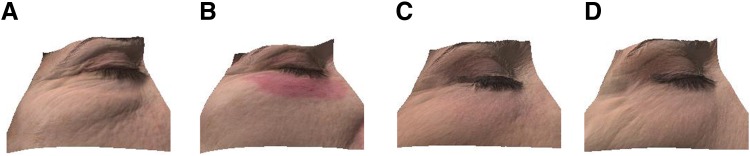
Antera 3D plots of a 52-year-old woman **(A)** pretreatment, **(B)** 7 days, **(C)** 1 month, and **(D)** 3 months post-treatment.

The 3D imaging device confirmed the objective evaluation of the clinical response (Fig. 4A). A reduction of medium protrusions and depressions and an improved texture were observed on the cutaneous surface (Fig. 4B–D). Further, marked increase in hemoglobin levels was visualized at 7 days postoperative compared with pretreatment conditions (Fig. 4E). Pretreatment levels were restored after 1 month from the procedure, suggesting a progressive recovery of the target zone. A 3D improvement was also observed (Fig. 5).

## Discussion

Nonsurgical treatment options for skin rejuvenation are increasingly requested by patients because of the reduced recovery times and complications often associated with more invasive surgery.^14,15^ Periorbital skin rejuvenation procedure requests follow the same trend.^3,4^ CO_2_ laser has shown a wide range of applications. In detail, its efficacy and safety have been shown in the treatment of scars and skin rejuvenation, as well as for other applications.^5–7,16,17^

This study proposes the freehand CO_2_ laser RAP technique for lower eyelid rejuvenation and outlines results in a cohort of 20 female patients.

In the current pilot study, the RAP technique has been demonstrated to be effective and safe. An improvement of the target area was observed in all patients, as assessed by blinded evaluations performed by external dermatologists, GAIS scores, patient satisfaction, and 3D imaging. The low pain scores and minor adverse events highlighted the technique's safety. In particular, edema resolves within 2–3 weeks, followed by the resolution of inflammation and heterogeneous color. These variations are documented by means of a 3D device. This device enables an *in vivo* observation, monitoring of skin texture, and an objective demonstration of skin changes in real time, reducing interobserver variability. The 3D imaging in this study highlights a general homogenization of skin surface, texture, and protrusions. Additionally, a reduction in edema and erythema following the peak observed at 7 days postintervention was observed with the same change in hemoglobin levels. The hemoglobin levels underline the presence of an inflammatory response, which was resolved after 30–60 days. This is important information for patients as a complete recovery can only be expected 1 month after the procedure.

Interestingly, clinical features of the skin of patients treated with the RAP technique (consisting of precise vaporization of the epidermis and minimal coagulation of the dermis) seem pivotal to the successful results, although it is a small population. Our data show that elastosis and atrophic skin of the lower eyelid, eventually combined with evidence of nasojugal fold, represent appropriate indications for the RAP technique. Additionally, these initial data suggest that the nasojugal fold (observed in six patients) can be corrected in all cases while respecting the proportion of the face (Figs. 3C, D, and 5). Therefore, neither filler injection nor fat transplant is additionally required.

In the last few years, other noninvasive techniques have been proposed for lower eyelid rejuvenation.^3,18–20^ However, these techniques, among which are microablative fractional laser, peelings, neuromodulators, and radiofrequency, can be potentially ineffective despite the necessity of multiple sessions of treatments. CO_2_ microablative fractional laser has been developed to deliver controlled epidermal and dermal damage to achieve wound healing and remodeling with a short downtime.^16,17^ Nevertheless, the application of fractional technology to eyelids has shown some limitations. The fractional laser employs higher energy (15–18 W), compared with RAP (0.5 W), since a part of this energy is absorbed from the epidermis before reaching the dermis. On the other hand, RAP exploits energy for retraction in specific areas of the superficial dermis directly after homogeneous and selective epidermal vaporization, following clinical color indicators. Bonan et al.^3^ performed fractional laser on both upper and lower eyelids at the same time. Consequently, global esthetic improvement of both eyelids has been estimated, without specific focus on the lower eyelid. In detail, 8.9% of the total 45 patients enrolled were not very satisfied with results obtained with the fractional laser. Further, compared with the fractional CO_2_ laser,^3^ no crusting was reported with RAP.

**FIG. 6.**
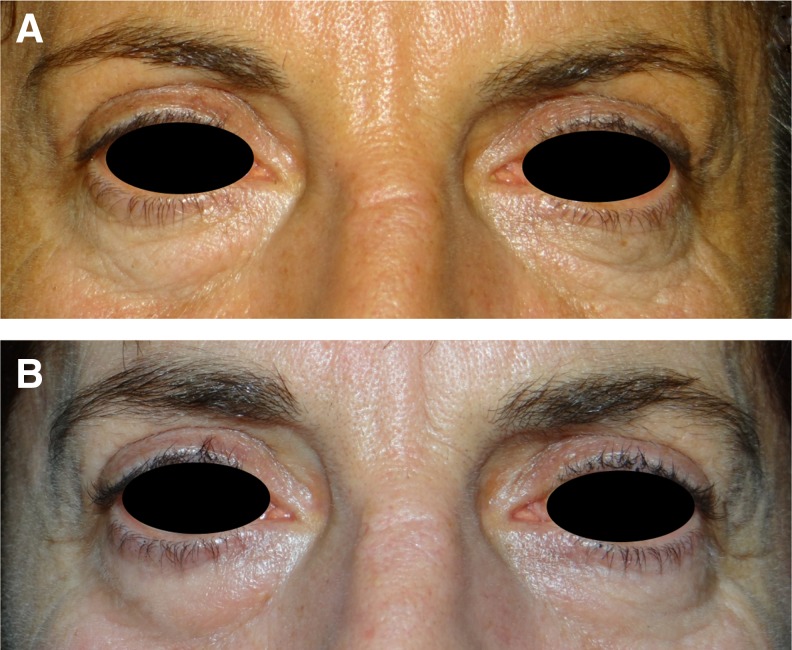
Clinical picture of a 54-year-old patient **(A)** pretreatment and **(B)** 9 months post-treatment.

The RAP technique can also be combined with blepharoplasty, with a 6-month interval between the two procedures. Interestingly, the trend of modern lower lid blepharoplasty focuses on minimal skin excision to remove fat bags to minimize complications. Consequently, eyelid laxity can occur, following minimal skin resection, thus requiring an integrated approach to maximize results. Some experiences of the combination of blepharoplasty with peelings have been described, but the peeling procedure needs to be repeated,^18,19^ RAP could represent an alternative integrated approach to transconjuntival blepharoplasty. It can be performed in a single sitting, with documented results lasting over 9 months (Fig. 6).

In addition, radiofrequency devices and neuromodulators (botulinum toxin) can achieve periorbital rejuvenation and dynamic rhytide improvement, respectively, without specific action on elastosis or atrophic skin of the lower eyelids.^20^

Taken together, our results demonstrate that expert dermatologists with laser experience can use RAP to maximize esthetic results that can be obtained with the CO_2_ laser technique and patient satisfaction while reducing complications usually associated with ablative laser treatment procedures. We highlight the importance of a learning curve to perform a correct procedure. As a matter of fact, too superficial or too deep vaporization should be avoided to obtain maximum efficacy and avoid scarring, respectively. The small number of patients enrolled, the lack of control group, and the absence of pre- and post-treatment histopathology examination limit the conclusions we can derive from this study. Nevertheless, this pilot study suggests that this new technique may be effective and safe for lower eyelid rejuvenation, as shown by clinical results. Further studies will be performed to confirm these data and to compare this technique with other available techniques.

## Conclusions

In the current pilot study, the RAP technique seems to be an effective and safe minimally invasive technique for rejuvenation of skin elastosis of the lower eyelid, with or without evidence of the nasojugal fold and atrophic and dyschromic skin. This small cohort is presented as single interventions and as an integrated approach for transconjuntival lower blepharoplasty. The 3D imaging tool's quantitative assessment confirmed the positive results, observed by qualitative evaluations, and estimated recovery time.
